# Pharmacological Management of Delirium in Older Adults in the Emergency Department: Clinical Outcomes

**DOI:** 10.3390/diseases14020068

**Published:** 2026-02-12

**Authors:** Angela Soler-Sanchis, Francisco Miguel Martínez-Arnau, Pilar Pérez-Ros

**Affiliations:** 1 Department of Nursing, Faculty of Nursing and Podiatry, Universitat de València, 46010 Valencia, Spain; angela.soler@uv.es (A.S.-S.); maria.p.perez-ros@uv.es (P.P.-R.); 2 Emergency Department, Francesc de Borja Hospital, 46702 Gandia, Spain; 3 Department of Physiotherapy, Universitat de València, Gascó Oliag 5, 46010 Valencia, Spain

**Keywords:** aged, delirium, drug therapy, emergency service, hospital, hospitalisation

## Abstract

**Background/Objectives:** Delirium is frequent and serious in older adults attending the emergency department (ED), but evidence on its pharmacological management in this setting is limited. This study aimed to quantify the pharmacological treatment of delirium in older adults in the ED and examine its association with subsequent hospital admission. **Methods:** A cross-sectional study was conducted between November 2021 and June 2022 in a Spanish ED. The sample included 153 adults aged 65 years or older with clinician-diagnosed delirium. Clinical, triage, and medication data were obtained from electronic medical records, and associations with hospital admission were analysed using multivariable logistic regression. **Results:** Ninety-one participants (59.5%) were hospitalised. Antipsychotic, analgesic, and benzodiazepine use was associated with hospitalisation. Absence of an underlying cause was a protective factor. The logistic regression model was significant. **Conclusions:** By identifying the most frequently administered pharmacological treatments for delirium in older adults in the ED and describing their association with hospitalisation, this study provides key insights into real-world clinical practice patterns in this setting.

## 1. Introduction

Delirium is an acute, prevalent, and severe neuropsychiatric syndrome characterised by rapid and fluctuating disturbances of attention, cognition, and consciousness [1]. Although it frequently occurs in the context of underlying medical conditions, it should not be understood merely as a clinical manifestation of these entities, but rather as a distinct syndrome with its own multifactorial pathophysiology [2]. In older adults, its incidence in the Emergency Department (ED) ranges from 7% to 20%, reaching up to 89% in people with previous cognitive impairment or dementia [3]. In around two-thirds of cases, delirium is not detected in the ED. The detection of hyperactive delirium is usually more straightforward because it presents visible and disruptive symptoms, such as agitation, restlessness, aggression, or hallucinations, which quickly alert healthcare staff. In contrast, hypoactive delirium manifests with lethargy, apathy, reduced speech, and decreased psychomotor activity. Because these symptoms are also indicative of depression, fatigue, frailty, and typical changes associated with hospitalisation in older adults, hypoactive delirium often goes unnoticed [4]. The presence of delirium is associated with increased morbidity, mortality, and functional impairment, and the worst outcomes occur in cases of unrecognised delirium. This underscores the importance of early detection and management [5].

The ED setting presents particular challenges for the diagnosis and treatment of delirium, due to the variability of signs and symptoms, high care burden, and limited care times [6]. The Manchester Triage System (MTS) is commonly used in EDs to assess clinical features with standardised flow charts and discriminators, and to classify patients according to priority levels PI (immediate, red), PII (very urgent, orange), PIII (urgent, yellow), PIV (standard, green), and PV (non-urgent, blue) [7]. Despite its role in structuring ED workflow, the MTS lacks specific protocols for detecting delirium. This deficiency, coupled with the syndrome’s fluctuating nature, contributes to underdiagnosis rates as high as 80% [8]. Detection of delirium in the ED relies primarily on the use of standardised screening tools, with the 4AT and bCAM being among the most widely validated and suitable instruments for acute care [9]. The 4AT has proven to be a highly sensitive, rapid, and feasible triage tool, enabling the prompt identification of delirium without increasing triage time in older adults [10]. Meanwhile, the bCAM is widely recommended in emergency settings as a confirmatory diagnostic tool and is included in the Geriatric Emergency Department Guidelines [9]. Systematic implementation of these scales is essential, as evidence shows that a significant proportion of delirium cases continue to go unrecognised by ED staff [4].

The management of delirium involves a combination of pharmacological and nonpharmacological strategies. Several international clinical guidelines, such as those produced by the American Geriatrics Society [11], the American Society for Enhanced Recovery [12], and the European Society of Anaesthesiology [13], advise against using antipsychotics and benzodiazepines in the older population because they are associated with important adverse events and prolonged confusion [14]. Nonetheless, these drugs are still widely administered in clinical practice, despite a lack of robust evidence supporting their efficacy and safety, especially in the hospital ED setting.

Current prevention and treatment strategies for delirium are based on studies from intensive care, surgical, internal medicine, or palliative care settings. Consequently, their applicability to the ED remains uncertain, and specific data for this context are scarce [15]. Furthermore, a lack of standardised clinical criteria means drug selection and dosing for delirium is often guided by individual experience rather than structured protocols, increasing the risk of overdose, adverse events, and suboptimal clinical outcomes [16]. This situation reflects a critical knowledge gap regarding the pharmacological management of delirium in the ED [17], where over half of geriatric hospitalisations originate. The management of delirium focuses primarily on identifying the underlying organic cause that triggers its onset, such as infection, metabolic disturbances, trauma, central nervous system disorders, or environmental factors. In the ED setting, even when the underlying cause is identified, or while it is being investigated, medications such as antipsychotics and benzodiazepines are often used for symptom control, a practice that is commonly associated with hyperactive delirium [18].

To our knowledge, no studies have specifically analysed the pharmacological treatment administered to older people with delirium in the ED. Therefore, the aim of this study was to quantify and characterise the pharmacological treatment used in this population and to analyse its association with hospitalisation. We sought to describe real-world treatment patterns that could inform future research in this clinical context.

## 2. Materials and Methods

### 2.1. Design

This was a cross-sectional study conducted between November 2021 and June 2022.

### 2.2. Study Setting and Sampling

The study was carried out in the Emergency Department (ED) of a tertiary care university hospital in eastern Spain. The centre has 256 beds, covering a population of about 188,000 inhabitants. The ED deals with an average of 60,000 episodes per year and is organised into six care areas: triage, admission, consultations, resuscitation, observation, and treatment room, as well as specific areas such as paediatrics and traumatology. The care circuit begins with triage by the nursing staff. Depending on the priority assigned, the doctor in charge carries out a clinical assessment, orders the relevant diagnostic tests, and prescribes the most appropriate treatment. The nursing staff then prepare and administer the treatment.

The prevalence of delirium among the 8426 individuals aged 65 years or older who visited the ED between November 2020 and June 2021 (one year prior to the study period) was 1.87%. Using this reference population, the minimum sample size was calculated assuming a 95% confidence level, 5% precision, and a minimum expected error of 2.5%, resulting in a required sample of 127 participants. After accounting for an anticipated 5% loss to follow-up, the final target sample size was adjusted accordingly.

### 2.3. Inclusion and Exclusion Criteria

The study population consisted of people aged 65 years or older who attended the ED between November 2021 and June 2022 and were diagnosed with delirium (International Classification of Diseases, tenth revision (ICD-10) code F05). These criteria include impaired attention and alertness, acute onset and fluctuating course, additional cognitive impairment, and exclusion of causes such as pre-existing neurocognitive disorders or severely decreased level of consciousness [19]. We excluded cases where delirium was clearly due to alcohol or substance abuse.

### 2.4. Data Collection

We collected the following data from participants’ electronic medical records during ED care: sociodemographic variables, MTS flow charts, priority assigned according to triage, underlying pathology associated with delirium, discharge destination, place of hospitalisation (if hospitalised), and pharmacological treatment administered, including the active ingredient, administration dose, and total number of drugs per patient.

### 2.5. Data Analysis

All data were reviewed and verified by an independent evaluator to ensure statistical quality and reliability. We performed a descriptive analysis of the sample, expressing continuous variables as means and standard deviations (SDs), and categorical variables as absolute frequencies and percentages.

To measure the association of each variable with hospital admission, we calculated odds ratios (ORs) and their respective 95% confidence intervals (CIs), and mean differences (MDs) with 95% CIs for continuous variables. Results with a *p* value below 0.05 were considered statistically significant.

We constructed a binary logistic regression model using backward stepwise selection, progressively eliminating variables that did not contribute significantly to the model fit, either because they did not substantially modify the estimated effect (variation < 10%) or because they did not improve the standard error. The model included variables with significance in the bivariate analysis and others considered clinically relevant according to the literature, such as MTS priority level, older age (>80 years), male sex, presence of an underlying disease, and pharmacological treatment administered in the ED (analgesics, antipsychotics, benzodiazepines, antibiotics, antidepressants, corticosteroids, antihypertensives, diuretics, anticonvulsants, antiarrhythmics, bronchodilators, and antidiabetics). Where several subsets of variables showed equal predictive capacity, we reached consensus through discussion for the final model selection. We determined the discriminative capacity of the model by analysing the receiver operating characteristic (ROC) curve, calculating the area under the curve (AUC) with its 95% CI as a performance indicator. All analyses were performed with IBM SPSS Statistics software for Windows, version 28.0 (IBM Corp., Armonk, NY, USA).

### 2.6. Ethical Considerations

The study was approved by the Research, Teaching, and Ethics Committee of the Department of Health of Gandía (approval number 15/2021) on 11 October 2021. All participants signed an informed consent form prior to inclusion. The research was carried out in accordance with the ethical principles of the Declaration of Helsinki and with Spanish Organic Law 3/2018, of 5 December, on Personal Data Protection and the Guarantee of Digital Rights. Data confidentiality was ensured at all times.

## 3. Results

During the study recruitment period, 53,110 people were seen in the ED of Francesc de Borja Hospital, of whom 28.6% (15,197) were over 65 years of age. Within this age group, 153 people (1.01%) had a diagnosis of delirium and formed our study sample. Ninety-one participants (59.5%) required hospitalisation, mainly in Internal Medicine (94.5%, n = 86), followed by urology and surgery (2.2%, n = 2 in each department) and the intensive care unit (1.1%, n = 1) (Table 1).

The mean age of our sample was 84 years, and there was a slight predominance of males. Most participants were classified as priority III (urgent; 62.1%, n = 95) and priority IV (standard; 31.4%, n = 48) in the MTS triage. The most commonly used MTS flow charts were ‘unwell adult’ (54.9%, n = 84), followed by ‘behaving strangely’ (15.0%, n = 23). Regarding associated clinical diagnoses, 68 participants (44.4%) had no underlying pathology associated with the delirium episode. However, 34 people (22.2%) had an infection, and 16 (10.5%) had kidney disease (Table 1).

Regarding pharmacological treatment, participants received a mean of 2.1 drugs (SD = 1.98), and the mean difference (MD) between hospitalised versus non-hospitalised participants was 1.97 drugs (95% CI 1.41 to 2.53; *p* < 0.001). Of note, 73.6% (n = 67) of participants who required hospital admission were treated with more than two active ingredients during their stay in the ED, compared with 16 participants (25.8%) of non-hospitalised participants (*p* < 0.001).

Finally, analysis of the pharmacological groups revealed that hospitalised participants were more frequently treated with antipsychotics (OR 9.93, 95% CI 3.32–29.73; *p* < 0.001), benzodiazepines (OR 5.00, 95% CI 1.08–23.00; *p* = 0.024), analgesics (OR 4.97, 95% CI 2.46–10.02; *p* < 0.001), and antibiotics (OR 4.15, 95% CI 1.92–8.97; *p* < 0.001), as shown in Table 1.

Analgesics that showed a statistically significant association with hospital admission were metamizole (OR 7.53, 95% CI 0.93–60.42; *p* = 0.028) and paracetamol (OR 3.35, 95% CI 1.66–6.77; *p* < 0.001), while ketamine showed a weaker association (OR 3.27, 95% CI 0.89–12.02; *p* = 0.061). Among the antipsychotics, there were associations for haloperidol (OR 5.19, 95% CI 1.46–18.39; *p* = 0.006) and tiapride (OR 3.57, 95% CI 1.14–11.14; *p* = 0.021). Finally, the benzodiazepine category showed an overall significant association with hospitalisation (OR 5.00, 95% CI 1.08–23.00; *p* = 0.024) (Table 2).

An analysis of the relationship between treatment and delirium aetiology showed that people with underlying pathologies such as infections, kidney disease, or respiratory disease were more likely to receive analgesics (*p* = 0.028) and antibiotics (*p* < 0.001) compared with people who had no apparent cause of delirium. There were also relevant differences in the use of corticosteroids, diuretics, antiulcer drugs, and bronchodilators, suggesting a therapeutic adaptation according to the clinical context of the patient. These findings reflect a more targeted treatment pattern depending on the clinical origin of delirium (Table 3).

**Figure 1:**
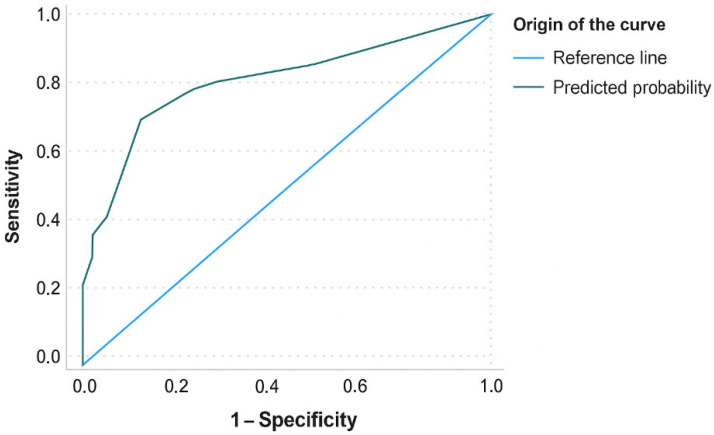
ROC curve for logistic regression model.

The logistic regression model constructed to predict hospitalisation in older adults with delirium was statistically significant (χ^2^ = 49.59, *p* < 0.001) with a Nagelkerke R^2^ of 0.374. It showed a sensitivity of 81.3% and a specificity of 66.1% and classified 59.5% of cases correctly. Variables included in the model were analgesics (OR 3.80, 95% CI 1.75–8.27; *p* < 0.001), antipsychotics (OR 9.61, 95% CI 3.02–30.56; *p* < 0.001), benzodiazepines (OR 3.93, 95% CI 0.75–20.5; *p* = 0.104), as well as the absence of an underlying cause, which behaved as a protective factor (OR 0.45, 95% CI 0.21–0.97; *p* = 0.043). The AUC was 0.80 (95% CI 0.72–0.86), indicating good discriminative ability to identify patients who were hospitalised (Figure 1).

## 4. Discussion

Previous evidence suggests that delirium in older people treated in the ED is associated with longer hospital stays, increased mortality, more clinical complications, a higher probability of discharge to institutional centres, and a considerable increase in the burden of care [20]. This study aimed to describe the clinical and pharmacological profile of older adults with delirium attending the ED and to analyse the association of these characteristics with hospitalisation. The prevalence of hospitalisation in our sample was 59.5%. The variables associated with admission included certain drug families, particularly analgesics, antipsychotics, and benzodiazepines.

The mean age observed in our sample (84 years) suggests a possible tendency towards the presentation of delirium in older patients compared to what is reported in the literature, where averages range from 71.8 years to 77 years [21]. This difference could be related to population-specific characteristics. On the other hand, the similar distribution of men and women in our sample is consistent with previous research, reinforcing the idea that neither sex has a greater predisposition to delirium [22].

Findings related to the MTS reinforce concerns raised about the sensitivity and suitability of this tool in older adults [23]. The high proportion of cases classified as urgent (62.1%) and standard priority (31.4%), together with the frequent use of the “unwell adult” (54.9%) and “behaving strangely” (15.0%) flow charts, suggests a possible underestimation of clinical severity. These results align with previous studies in geriatric populations and suggest that the MTS may have systematic limitations in recognising the clinical complexity of older adults, particularly in conditions like delirium [24]. The incorporation of validated delirium screening tools (4AT, bCAM DTS) in the ED is essential to improve early and accurate detection of this condition by providing objective, standardised criteria, reducing the likelihood of missed cases. Implementing these validated scales systematically can therefore improve patient outcomes, guide timely interventions, and ensure safer, more effective emergency care [25].

No underlying cause of delirium was identified in 35.2% of hospitalised people in our sample. This highlights the diagnostic difficulties of delirium in the ED setting, especially among older adults [26]. The multifactorial pathophysiology of delirium, together with precipitating factors such as pain, immobilisation, and polypharmacy, can preclude establishing a clear aetiology in the initial assessment [27]. In cases where clinicians identified a specific cause, the cause most frequently associated with hospitalisation was infection (30.8% in hospitalised participants vs. 9.7% in non-hospitalised participants). This is likely because infections in older adults can have a substantial physiological impact and often manifest atypically, with delirium as the primary symptom. Hospital admission is necessary in these cases for proper assessment and treatment [28].

Our analysis of drug consumption showed that participants who required hospitalisation received a higher number of drugs in the ED than non-hospitalised participants (MD 1.97 drugs, 95% CI 1.41–2.53; *p* < 0.001), suggesting a possible association between the number of drugs administered in the ED and the clinical severity or complexity of the condition. This pattern is consistent with the literature, which reports that people with delirium are usually treated with one or two active ingredients [29]. Recent studies have described ED administration of antipsychotics (e.g., haloperidol and olanzapine), benzodiazepines (e.g., midazolam and lorazepam) [30], ketamine, opioids, and alpha-2 agonists such as dexmedetomidine [31].

Antipsychotics can be useful in treating acute mental disorders, although the evidence for their efficacy and safety in people with delirium remains limited and controversial, with some authors warning of potential risks such as increased mortality, prolonged symptoms, and serious adverse effects [32]. In our sample, antipsychotic use was significantly associated with hospitalisation, probably because these drugs are reserved for the most severe and complex cases [33]. Although several studies have evaluated the use of antipsychotics in settings with a high prevalence of delirium, such as intensive care units, general hospital wards, postoperative care, palliative care, and rehabilitation departments, there is a lack of evidence guiding their application in EDs, underscoring the need for more prospective research [34].

Given that haloperidol and tiapride were the antipsychotics that showed a significant association with hospitalisation in our study, it is pertinent to analyse them in greater depth. The prophylactic use of haloperidol in older people is not recommended due to a lack of solid evidence supporting its efficacy in preventing or managing delirium [35]. However, the coadministration of haloperidol with other drugs, such as lorazepam, has shown better response rates in specific clinical contexts [36]. Regarding tiapride, its frequent use in the ED contrasts with its scarce presence in the international scientific literature. This disparity could reflect local clinical practices or non-standardised therapeutic decisions, highlighting the need for further research into the potential benefits of atypical antipsychotics in delirium management. To date, very few studies have specifically addressed the use of this drug, so our findings constitute a novel contribution and open a little-explored line of research [37].

In our sample, people who received benzodiazepines were more frequently hospitalised. This may be due to several factors: on the one hand, benzodiazepines may be administered in cases of severe hyperactive delirium, which would imply a higher clinical risk and justify admission regardless of the treatment used; and on the other hand, their effects on the sleep–wake cycle could contribute to worsening the condition, which in turn would require a longer hospital stay [38]. The evidence on the use of benzodiazepines is contradictory and depends largely on the clinical context in which it is evaluated. In intensive care units, most studies advise against their use due to an association with worsening delirium [39]. In contrast, benzodiazepines are used for delirium prevention and treatment in the perioperative setting, although the results are inconsistent [40]. Some systematic reviews and meta-analyses have found no difference in the incidence of postoperative delirium following their use, while other studies have described an increased risk attributed to paradoxical effects such as irritability and confusion, especially with repeated doses [41]. In the ED setting, despite the potential usefulness of benzodiazepines in specific clinical situations such as agitation [42] or withdrawal syndrome [43], there is still little evidence focusing on their safety and efficacy in cases of delirium.

In this study, there was an association between analgesic use and the need for hospitalisation. Pain is not only a symptom but also a precipitating factor for delirium when poorly managed in older people. Therefore, the administration of analgesics could be interpreted as an indirect marker of greater clinical severity or an underlying cause that would justify hospitalisation [44]. Evidence on the specific impact of different analgesics on the progression of delirium remains limited and heterogeneous [45]. The analgesic most frequently associated with hospitalisation in our study was metamizole, a finding that is difficult to interpret in the absence of previous studies. It is possible that this relationship reflects the presence of pain as a symptom of clinical severity or as a precipitating factor for delirium, rather than an effect of the drug [44]. Paracetamol, widely used for mild to moderate pain, was also associated with higher admission rates in our sample, likely due to its recognised safety and frequent use in the older population. Finally, ketamine has been explored as an alternative analgesic in EDs for refractory pain. Although some studies have found no direct link between ketamine use and the development of delirium, its role remains controversial due to psychotropic effects and the limited evidence available [46].

Pharmacological treatment plays a key role in managing numerous acute conditions (e.g., pain, agitation, or withdrawal syndromes) that require rapid and effective intervention in the ED. However, a particularly careful approach is necessary when these conditions are aetiologically or symptomatically related to delirium [47]. In addition, family involvement and non-pharmacological care strategies have been identified as key components in delirium management in critical care settings, further underscoring the multifactorial nature of delirium and the need to adapt these approaches to the ED context [48]. In this setting, the inappropriate or non-individualised use of certain drugs can have significant adverse consequences, including hospitalisation, a longer hospital stay, or an increased mortality risk [49]. Our results show that the administration of antipsychotics, analgesics, and benzodiazepines in the ED is associated with hospital admission in older people with delirium. This pharmacological profile may be associated with greater clinical complexity and may help to inform future research on delirium management in the ED. Furthermore, these findings highlight the need for further studies to better understand the relationship between pharmacological management and hospital admission.

This study has limitations inherent to its single-centre, cross-sectional design, which restricts the generalisability of the findings and precludes causal inference. In addition, it was not possible to collect some potentially relevant variables, such as delirium severity, length of stay in the ED, or the exact timing of drug administration, which may have acted as confounding factors. Future studies should address these limitations through prospective, multicentre designs and evaluate specific pharmacological and non-pharmacological interventions in the ED setting.

## 5. Conclusions

This study quantifies the most frequent pharmacological management of delirium in older adults in the emergency department and identifies associations between hospitalisation and the use of antipsychotics, benzodiazepines, and analgesics. These findings highlight patterns of real-world pharmacotherapy in this clinical setting and may help to contextualise the management of delirium in older patients. Further studies are needed to better understand the clinical implications of these associations.

### Relevance for Clinical Practice

This cross-sectional study provides a pioneering characterisation of the pharmacological management of delirium in older adults. Understanding which pharmacological prescriptions are associated with a higher likelihood of hospital admission provides an important starting point for interpreting the relationship between symptom severity and the probability of inpatient care. Our findings suggest that more severe or disruptive clinical presentations—typically characteristic of hyperactive delirium—are more readily identified and therefore more likely to lead to hospitalisation, while hypoactive forms remain underdiagnosed due to their subtle and often misinterpreted manifestations. Furthermore, our findings reinforce the need for systematic identification of the underlying organic cause of delirium, as the precipitating condition often goes unrecognised in the ED, contributing to missed or delayed diagnosis.

## Figures and Tables

**Table 1:** Participant characteristics according to hospital admission.

	Hospitalisation % (n) ^1^		Total % (n) ^1^	OR (95% CI)	*p* Value ^2^
Variable	Yes (n = 91)	No (n = 62)	(n = 153)		
Age, years, mean (SD)	83.82 (7.76)	84.35 (7.60)	84.04 (7.68)		0.605
Total drugs, mean (SD)	2.92 (2.02)	0.95 (1.16)	2.12 (1.98)		<0.001
MTS diagram					
Unwell adult	51.6 (47)	59.7 (37)	54.9 (84)		0.572
Behaving strangely	14.3 (13)	16.1 (10)	15.0 (23)		
Shortness of breath	7.7 (7)	9.7 (6)	8.5 (13)		
Urinary problems	3.3 (3)	4.8 (3)	3.9 (6)		
Mental illness	2.2 (2)	1.6 (1)	2.0 (3)		
Limb problems	13.2 (12)	3.2 (2)	9.2 (14)		
Abdominal pain	4.4 (4)	1.6 (1)	3.3 (5)		
Infections	1.1 (1)	0	0.7 (1)		
Other	2.2 (2)	3.2 (2)	2.6 (4)		
Clinical diagnosis					
No underlying disease	35.2 (32)	58.1 (36)	44.4 (68)		0.012
Cardiac disease	5.5 (5)	0 (0)	3.3 (5)		
Neurological disease	7.7 (7)	12.9 (8)	9.8 (15)		
Renal disease	11 (10)	9.7 (6)	10.5 (16)		
Respiratory disease	5.5 (5)	6.5 (4)	5.9 (9)		
GI disease	4.4 (4)	3.2 (2)	3.9 (6)		
Infection	30.8 (28)	9.7 (6)	22.2 (34)		
Sex					
Male	50.5 (46)	50 (31)	50.3 (77)	0.97 (0.51–1.86)	0.947
Female	49.5 (45)	50 (31)	49.7 (76)	0.98 (0.72–1.36)	0.539
MTS priority					
Priority IV (standard)	25.3 (23)	40.3 (25)	31.4 (48)	0.50 (0.25–1.00)	0.049
Priority III (urgent)	68.1 (62)	53.2 (33)	62.1 (95)	1.88 (0.96–3.65)	0.062
Priority II (very urgent)	6.6 (6)	6.5 (4)	6.5 (10)	1.02 (0.27–3.78)	0.972
Treatment					
≥2 active ingredients	73.6 (67)	25.8 (16)	54.2 (83)	8.02 (3.84–16.74)	<0.001
Analgesics	67.0 (61)	29 (18)	51.6 (79)	4.97 (2.46–10.02)	<0.001
Antiemetics	5.5 (5)	0	3.3 (5)	1.06 (1.00–1.11)	0.061
Antipsychotics	40.7 (37)	6.5 (4)	26.8 (41)	9.93 (3.32–29.73)	<0.001
Benzodiazepines	14.3 (13)	3.2 (2)	9.8 (15)	5.00 (1.08–23.00)	0.024
Antibiotics	47.3 (43)	17.7 (11)	35.3 (54)	4.15 (1.92–8.97)	<0.001
Corticosteroids	8.8 (8)	4.8 (3)	7.2 (11)	1.89 (0.48–7.44)	0.353
Antidepressants	8.8 (8)	0	5.2 (8)	0.57 (0.49–0.65)	0.016
Antihypertensives	2.2 (2)	0	1.3 (2)	0.58 (0.51–0.67)	0.240
Diuretics	11.0 (10)	3.2 (2)	7.8 (12)	3.07 (0.78–17.52)	0.080
Anti-ulcer drugs	5.5 (5)	1.6 (1)	3.9 (6)	3.54 (0.40–31.12)	0.225
Anticonvulsants	4.4 (4)	1.6 (1)	3.3 (5)	2.80 (0.30–25.71)	0.342
Antiarrhythmics	2.2 (2)	0	1.3 (2)	0.58 (0.51–0.67)	0.240
Antidiabetics	2.2 (2)	1.6 (1)	2.0 (3)	1.37 (0.12–15.45)	0.798
Bronchodilators	2.2 (2)	3.2 (2)	2.6 (4)	0.67 (0.09–4.91)	0.696
Antiplatelets	8.8 (8)	0	5.2 (8)	0.57 (0.49–0.65)	0.016
Others	2.2 (2)	3.2 (2)	2.6 (4)	0.67 (0.09–4.91)	0.696

^1^ Unless otherwise specified in the Variable column. ^2^ Chi-squared *p* values for categorical variables and Mann–Whitney U *p* values for continuous variables (age and total drugs administered). Abbreviations: CI, confidence interval; GI, gastrointestinal; MTS, Manchester Triage System; OR, odds ratio.

**Table 2:** Analysis of active ingredients for the most representative families of drugs related to hospital admission.

	Hospitalisation % (n)		Total % (n)	OR (95% CI)	*p* Value ^1^
	Yes (n = 91)	No (n = 62)	(n = 153)		
Analgesics	67.0 (61)	29.0 (18)	51.6 (79)	4.97 (2.46–10.02)	<0.001
Metamizole (≥2 g)	11.0 (10)	1.6 (1)	7.2 (11)	7.53 (0.93–60.42)	0.028
Paracetamol (≥1 g)	53.8 (49)	25.8 (16)	42.5 (65)	3.35 (1.66–6.77)	<0.001
Dexketoprofen (≥50 mg)	2.2 (2)	1.6 (1)	2.0 (3)	1.37 (0.12–15.54)	0.798
Tramadol (≥100 mg)	3.3 (3)	1.6 (1)	2.6 (4)	2.08 (0.21–20.46)	0.522
Ketamine	14.3 (13)	4.8 (3)	10.5 (16)	3.27 (0.89–12.02)	0.061
<50 mg	14.3 (13)	3.2 (2)	9.8 (15)	5.00 (1.09–23.00)	0.024
≥50 mg	0	1.6 (1)	0.7 (1)	0.98 (0.95–1.01)	0.972
Morphine	6.6 (6)	0	3.9 (6)	0.57 (0.50–0.66)	0.039
<50 mg	5.5 (5)	0	3.3 (5)	1.06 (1.00–1.11)	0.167
≥50 mg	1.1 (1)	0	0.7 (1)	1.01 (0.99–1.03)	0.408
Fentanyl (<150 mg)	1.1 (1)	0	0.7 (1)	0.59 (0.52–0.67)	0.408
Antipsychotics	40.7 (37)	6.5 (4)	26.8 (41)	9.93 (3.32–29.73)	<0.001
Haloperidol	20.9 (19)	4.8 (3)	14.4 (22)	5.19 (1.46–18.39)	0.006
<15 mg	19.8 (18)	4.8 (3)	13.7 (21)	4.85 (1.36–17.26)	0.008
≥15 mg	1.1 (1)	0	0.7 (1)	1.01 (0.99–1.03)	0.408
Olanzapine	2.2 (2)	0	1.3 (2)	0.59 (0.51–0.67)	0.240
<15 mg	1.1 (1)	0	0.7 (1)	1.01 (0.99–1.03)	0.408
≥15 mg	1.1 (1)	0	0.7 (1)	1.01 (0.99–1.03)	0.408
Risperidone	2.2 (2)	0	1.3 (2)	0.58 (0.51–0.67)	0.240
<5 mg	1.1 (1)	0	0.7 (1)	1.01 (0.99–1.03)	0.408
≥5 mg	1.1 (1)	0	0.7 (1)	1.01 (0.99–1.03)	0.408
Tiapride	19.8 (18)	6.5 (4)	14.4 (22)	3.57 (1.14–11.14)	0.021
<100 mg	3.3 (3)	0	2.0 (3)	1.03 (0.99–1.07)	0.149
≥100 mg	16.5 (15)	6.5 (4)	12.4 (19)	2.86 (0.92–9.08)	0.065
Levomepromazine (≥125 mg)	1.1 (1)	0	0.7 (1)	0.59 (0.51–0.67)	0.408
Benzodiazepines	14.3 (13)	3.2 (2)	9.8 (15)	5.00 (1.08–23.00)	0.024
Diazepam (≥5 mg)	3.3 (3)	1.6 (1)	2.6 (4)	2.08 (0.21–20.46)	0.522
Alprazolam (≥1 mg)	2.2 (2)	0	1.3 (2)	0.58 (0.51–0.67)	0.240
Lorazepam (≥1 mg)	2.2 (2)	1.6 (1)	2.0 (3)	1.37 (0.12–15.45)	0.798
Midazolam	4.4 (4)	0	2.6 (4)	0.58 (0.51–0.67)	0.094
<3 mg	1.1 (1)	0	0.7 (1)	1.01 (0.99–1.03)	0.408
≥3 mg	3.3 (3)	0	2.0 (3)	1.03 (0.99–1.07)	0.149
Clorazepate (≥10 mg)	1.1 (1)	0	0.7 (1)	0.59 (0.51–0.67)	0.408
Bromazepam (≥1.5 mg)	1.1 (1)	0	0.7 (1)	0.59 (0.51–0.67)	0.408

^1^ Chi-squared *p* values. Abbreviations: CI, confidence interval; OR, odds ratio.

**Table 3:** Analysis of active ingredients for the most representative families of drugs related to the organic cause of delirium.

	No Underlying Disease ^1^ (n = 68)	Heart Disease ^1^ (n = 5)	Neurological Disease ^1^ (n = 15)	Kidney Disease ^1^ (n = 16)	Respiratory Disease ^1^ (n = 9)	GI Disease ^1^ (n = 6)	Infection ^1^ (n = 34)	Total ^1^ (n = 153)	*p* Value ^2^
Analgesics	39.7 (27)	60.0 (3)	33.3 (5)	62.5 (10)	55.6 (5)	83.3 (5)	70.6 (24)	51.6 (79)	0.028
Antiemetics	10.3 (7)	40.0 (2)	6.7 (1)	25.0 (4)	22.2 (2)	33.3 (2)	26.5 (9)	17.6 (27)	0.170
Antipsychotics	22.1 (15)	60.0 (3)	20.0 (3)	25.0 (4)	22.2 (2)	16.7 (1)	38.2 (13)	26.8 (41)	0.355
Antibiotics	0	80.0 (4)	13.3 (2)	68.8 (11)	66.7 (6)	33.3 (2)	58.3 (29)	35.3 (54)	<0.001
Corticosteroids	4.4 (3)	0	0	0	33.3 (3)	0	14.7 (5)	7.2 (11)	0.013
Antidepressants	2.9 (2)	20.0 (1)	0	12.5 (2)	0	0	8.8 (3)	5.2 (8)	0.306
Antihypertensives	1.5 (1)	0	6.7 (1)	0	0	0	0	1.3 (2)	0.639
Diuretics	1.5 (1)	60.0 (3)	0	12.5 (2)	22.2 (2)	16.7 (1)	8.8 (3)	7.8 (12)	<0.001
Anti-ulcer drugs	1.5 (1)	0	6.7 (1)	0	11.1 (1)	33.3 (2)	2.9 (1)	3.9 (6)	0.008
Benzodiazepines	10.3 (7)	0	13.3 (2)	12.5 (2)	0	0	11.8 (4)	9.8 (15)	0.847
Anticonvulsants	2.9 (2)	0	6.7 (1)	6.3 (1)	0	0	2.9 (1)	3.3 (5)	0.944
Antiarrhythmics	0	0	0	6.3 (1)	11.1 (1)	0	0	1.3 (2)	0.076
Antidiabetics	1.5 (1)	0	0	6.3 (1)	0	0	2.9 (1)	2.0 (3)	0.870
Bronchodilators	1.5 (1)	0	0	0	0	22.2 (2)	2.9 (1)	2.6 (4)	0.020
Antiplatelets	1.5 (1)	0	0	18.8 (3)	0	16.7 (1)	8.8 (3)	5.2 (8)	0.064
Others	2.9 (2)	0	6.7 (1)	0	0	0	2.9 (1)	2.6 (4)	0.922

^1^ Values presented as percentage (number) of participants. ^2^ Chi-square *p* values. Abbreviation: GI: gastrointestinal.

## Data Availability

The data from this study contain sensitive clinical information and, for ethical and privacy reasons, will not be made available in open repositories. However, they may be provided to qualified researchers upon reasonable request, as participants gave their consent for the use and transfer of their anonymized data for research purposes.

## Abbreviations

AUC	Area Under the Curve
CI	Confidence Interval
DSM-5	Diagnostic and Statistical Manual of Mental Disorders, Fifth Edition
ED	Emergency Department
GI	Gastrointestinal
ICD-10	International Classification of Diseases, Tenth Revision
MD	Mean Differences
MTS	Manchester Triage System
OR	Odds Ratio
ROC	Receiver Operating Characteristic
SD	Standard Deviation
SPSS	Statistical Package for the Social Sciences

## References

[1] Ayón-Aguilar J., Serrano-Vértiz L., Quiroz-Lara F.V., Torres-Macotela M. (2025). ncidence and factors associated with delirium in an Emergency Department. Rev. Med. Inst. Mex. Seguro Soc..

[2] Fan Y.-Y., Luo R.-Y., Wang M.-T., Yuan C.-Y., Sun Y.-Y., Jing J.-Y. (2024). Mechanisms Underlying Delirium in Patients with Critical Illness. Front. Aging Neurosci..

[3] Morandi A., Inzitari M., Udina C., Gual N., Mota M., Tassistro E., Andreano A., Cherubini A., Gentile S., Mossello E. (2021). Visual and Hearing Impairment Are Associated with Delirium in Hospitalized Patients: Results of a Multisite Prevalence Study. J. Am. Med. Dir. Assoc..

[4] Meged-Book T., Frenkel R., Nikonov A., Zeldetz V., Kosto A., Schwarzfuchs D., Freud T., Press Y. (2024). Delirium Screening in the Emergency Department: Evaluation and Intervention. Isr. J. Health Policy Res..

[5] Ní Chróinín D., Wang S., Nagaraj G., Ren S., Middleton P.M., Short A. (2025). A Pilot Trial Exploring the Use of Music in the Emergency Department and Its Association with Delirium and Other Clinical Outcomes. Emerg. Med. Australas..

[6] Joseph J.W., Elhadad N., Mattison M.L.P., Nentwich L.M., Levine S.A., Marcantonio E.R., Kennedy M. (2024). Boarding Duration in the Emergency Department and Inpatient Delirium and Severe Agitation. JAMA Netw. Open.

[7] Zaboli A., Sibilio S., Massar M., Brigiari G., Magnarelli G., Parodi M., Mian M., Pfeifer N., Brigo F., Turcato G. (2024). Enhancing Triage Accuracy: The Influence of Nursing Education on Risk Prediction. Int. Emerg. Nurs..

[8] Chary A.N., Bhananker A.R., Brickhouse E., Torres B., Santangelo I., Godwin K.M., Naik A.D., Carpenter C.R., Liu S.W., Kennedy M. (2024). Implementation of Delirium Screening in the Emergency Department: A Qualitative Study with Early Adopters. J. Am. Geriatr. Soc..

[9] Lee S., Khoujah D., Eagles D., Kennedy M., Lo A.X., Nickel C.H., Arendts G., Ragsdale L., Seidenfeld J., de Wit K. (2025). GRADE-Based Clinical Practice Guidelines for Emergency Department Delirium Risk Stratification, Screening, and Brain Imaging in Older Patients With Suspected Delirium. Acad. Emerg. Med..

[10] Soler-Sanchis A., Martínez-Arnau F.M., Sánchez-Frutos J., Pérez-Ros P. (2024). The 4AT Scale for Rapid Detection of Delirium in Emergency Department Triage. Front. Med..

[11] (2015). The American Geriatrics Society Expert Panel on Postoperative Delirium in Older Adults. American Geriatrics Society Abstracted Clinical Practice Guideline for Postoperative Delirium in Older Adults. J. Am. Geriatr. Soc..

[12] Hughes C.G., Boncyk C.S., Culley D.J., Fleisher L.A., Leung J.M., McDonagh D.L., Gan T.J., McEvoy M.D., Miller T.E. (2020). American Society for Enhanced Recovery and Perioperative Quality Initiative Joint Consensus Statement on Postoperative Delirium Prevention. Anesth. Analg..

[13] Aldecoa C., Bettelli G., Bilotta F., Sanders R.D., Aceto P., Audisio R., Cherubini A., Cunningham C., Dabrowski W., Forookhi A. (2024). Update of the European Society of Anaesthesiology and Intensive Care Medicine Evidence-Based and Consensus-Based Guideline on Postoperative Delirium in Adult Patients. Eur. J. Anaesthesiol..

[14] Zarour S., Weiss Y., Kiselevich Y., Iacubovici L., Karol D., Shaylor R., Davydov T., Matot I., Cohen B. (2024). The Association between Midazolam Premedication and Postoperative Delirium—A Retrospective Cohort Study. J. Clin. Anesth..

[15] Carpenter C.R., Hammouda N., Linton E.A., Doering M., Ohuabunwa U.K., Ko K.J., Hung W.W., Shah M.N., Lindquist L.A., Biese K. (2021). Delirium Prevention, Detection, and Treatment in Emergency Medicine Settings: A Geriatric Emergency Care Applied Research (GEAR) Network Scoping Review and Consensus Statement. Acad. Emerg. Med..

[16] Skains R.M., Hayes J.M., Selman K., Zhang Y., Thatphet P., Toda K., Hayes B.D., Tayes C., Casey M.F., Moreton E. (2025). Emergency Department Programs to Support Medication Safety in Older Adults: A Systematic Review and Meta-Analysis. JAMA Netw. Open.

[17] Zaher-Sánchez S., Satústegui-Dordá P.J., Ramón-Arbués E., Santos-Sánchez J.A., Aguilón-Leiva J.J., Pérez-Calahorra S., Juárez-Vela R., Sufrate-Sorzano T., Angulo-Nalda B., Garrote-Cámara M.E. (2024). The Management and Prevention of Delirium in Elderly Patients Hospitalised in Intensive Care Units: A Systematic Review. Nurs. Rep..

[18] Ehrlich A., Oh E.S., Ahmed S. (2024). Managing Delirium in the Emergency Department: An Updated Narrative Review. Curr. Geriatr. Rep..

[19] European Delirium Association, American Delirium Society (2014). The DSM-5 Criteria, Level of Arousal and Delirium Diagnosis: Inclusiveness is Safer. BMC Med..

[20] Al Huraizi A.R., Al-Maqbali J.S., Al Farsi R.S., Al Zeedy K., Al-Saadi T., Al-Hamadani N., Al Alawi A.M. (2023). Delirium and Its Association with Short- and Long-Term Health Outcomes in Medically Admitted Patients: A Prospective Study. J. Clin. Med..

[21] Billig A.E., Lampert M.A., Guerra R.R., Steigleder N.E. (2022). Delirium in the elderly admitted to an emergency hospital service. Rev. Bras. Enferm..

[22] Soler-Sanchis A., Martínez-Arnau F.M., Sánchez-Frutos J., Pérez-Ros P. (2023). Clinical Risk Group as a Predictor of Mortality in Delirious Older Adults in the Emergency Department. Exp. Gerontol..

[23] Li B., Zhang Z., Li K., Deng Y. (2024). The Effectiveness of a Modified Manchester Triage System for Geriatric Patients: A Retrospective Quantitative Study. Nurs. Open.

[24] Soler-Sanchis A., Martínez-Arnau F.M., Sánchez-Frutos J., Pérez-Ros P. (2022). Identification through the Manchester Triage System of the Older Population at Risk of Delirium: A Case-Control Study. J. Clin. Nurs..

[25] O’Sullivan D., Brady N., Manning E., O’Shea E., O’Grady S., O ’Regan N., Timmons S. (2018). Validation of the 6-Item Cognitive Impairment Test and the 4AT Test for Combined Delirium and Dementia Screening in Older Emergency Department Attendees. Age Ageing.

[26] Lee S., Howard M.A., Han J.H. (2023). Delirium and Delirium Prevention in the Emergency Department. Clin. Geriatr. Med..

[27] Lee S., Angel C., Han J.H. (2021). Succinct Approach to Delirium in the Emergency Department. Curr. Emerg. Hosp. Med. Rep..

[28] Anand A., Maclullich A. (2024). Delirium in Older Adults. Medicine.

[29] Bonfichi A., Ceresa I.F., Piccioni A., Zanza C., Longhitano Y., Boudi Z., Esposito C., Savioli G. (2023). A Lethal Combination of Delirium and Overcrowding in the Emergency Department. J. Clin. Med..

[30] Dahlstrom E.B., Han J.H., Healy H., Kennedy M., Arendts G., Lee J., Carpenter C., Lee S. (2020). Delirium Prevention and Treatment in the Emergency Department (ED): A Systematic Review Protocol. BMJ Open.

[31] Sun C., Hirata Y., Kawahara T., Kawashima M., Sato M., Nakajima J., Anraku M. (2025). Diagnosis of Respiratory Sarcopenia for Stratifying Postoperative Risk in Non-Small Cell Lung Cancer. JAMA Surg..

[32] Casey M.F., Elder N.M., Fenn A., Niznik J., Khoujah D., Cole J.B., Cardon Z., Ding M., Goukasian N., Moreton E. (2025). Comparative Safety of Medications for Severe Agitation: A Geriatric Emergency Department Guidelines 2.0 Systematic Review. J. Am. Geriatr. Soc..

[33] Carayannopoulos K.L., Alshamsi F., Chaudhuri D., Spatafora L., Piticaru J., Campbell K., Alhazzani W., Lewis K. (2024). Antipsychotics in the Treatment of Delirium in Critically Ill Patients: A Systematic Review and Meta-Analysis of Randomized Controlled Trials. Crit. Care Med..

[34] Hui D., Cheng S.-Y., Paiva C.E. (2024). Pharmacologic Management of End-of-Life Delirium: Translating Evidence into Practice. Cancers.

[35] Huang J., Zheng H., Zhu X., Zhang K., Ping X. (2023). The Efficacy and Safety of Haloperidol for the Treatment of Delirium in Critically Ill Patients: A Systematic Review and Meta-Analysis of Randomized Controlled Trials. Front. Med..

[36] Wu Y.-C., Tseng P.-T., Tu Y.-K., Hsu C.-Y., Liang C.-S., Yeh T.-C., Chen T.-Y., Chu C.-S., Matsuoka Y.J., Stubbs B. (2019). Association of Delirium Response and Safety of Pharmacological Interventions for the Management and Prevention of Delirium: A Network Meta-Analysis. JAMA Psychiatry.

[37] Zangani C., Giordano B., Stein H.-C., Bonora S., Ostinelli E.G., D’Agostino A. (2022). Efficacy of Tiapride in the Treatment of Psychiatric Disorders: A Systematic Review. Hum. Psychopharmacol..

[38] Vollmer N.J., Wieruszewski E.D., Nei A.M., Mara K.C., Rabinstein A.A., Brown C.S. (2024). Impact of Continuous Infusion Ketamine Compared to Continuous Infusion Benzodiazepines on Delirium in the Intensive Care Unit. J. Intensive Care Med..

[39] van der Hoeven A.E., Bijlenga D., van der Hoeven E., Schinkelshoek M.S., Hiemstra F.W., Kervezee L., van Westerloo D.J., Fronczek R., Lammers G.J. (2024). Sleep in the Intensive and Intermediate Care Units: Exploring Related Factors of Delirium, Benzodiazepine Use and Mortality. Intensive Crit. Care Nurs..

[40] Fenta E., Teshome D., Kibret S., Hunie M., Tiruneh A., Belete A., Molla A., Dessie B., Geta K. (2025). Incidence and Risk Factors of Postoperative Delirium in Elderly Surgical Patients 2023. Sci. Rep..

[41] Xue X., Ma X., Zhao B., Liu B., Zhang J., Li Z., Li H., Liu X., Zhao S. (2025). The Impact of Remimazolam Compared to Propofol on Postoperative Delirium: A Systematic Review and Meta-Analysis. Minerva Anestesiol..

[42] Simon E.L., Smalley C.M., Muir M., Mangira C.M., Pence R., Wahi-Singh B., Delgado F., Fertel B.S. (2023). Agitation Management in the Emergency Department with Physical Restraints: Where Do These Patients End Up?. West. J. Emerg. Med..

[43] Sullivan S.M., Dewey B.N., Jarrell D.H., Vadiei N., Patanwala A.E. (2019). Comparison of Phenobarbital-Adjunct versus Benzodiazepine-Only Approach for Alcohol Withdrawal Syndrome in the ED. Am. J. Emerg. Med..

[44] White N., Bazo-Alvarez J.C., Koopmans M., West E., Sampson E.L. (2024). Understanding the Association between Pain and Delirium in Older Hospital Inpatients: Systematic Review and Meta-Analysis. Age Ageing.

[45] Leong A.Y., Edginton S., Lee L.A., Jaworska N., Burry L., Fiest K.M., Doig C.J., Niven D.J. (2025). The Association between Pain, Analgesia, and Delirium among Critically Ill Adults: A Systematic Review and Meta-Analysis. Intensive Care Med..

[46] Riccardi A., Guarino M., Serra S., Spampinato M.D., Vanni S., Shiffer D., Voza A., Fabbri A., De Iaco F. (2023). Study and Research Center of the Italian Society of Emergency Medicine Narrative Review: Low-Dose Ketamine for Pain Management. J. Clin. Med..

[47] Ozga D., Krupa S., Witt P., Mędrzycka-Dąbrowska W. (2020). Nursing Interventions to Prevent Delirium in Critically Ill Patients in the Intensive Care Unit during the COVID19 Pandemic-Narrative Overview. Healthcare.

[48] Lange S., Mȩdrzycka-Da Browska W., Friganović A., Religa D., Krupa S. (2022). Family Experiences and Attitudes toward Care of ICU Patients with Delirium: A Scoping Review. Front. Public Health.

[49] Jia B., Zhou S., Li J., Wan L., Zhou Y., Cui Y. (2025). Risk of Drug-Induced Delirium in Older Patients- a Pharmacovigilance Study of FDA Adverse Event Reporting System Database. Expert. Opin. Drug Saf..

